# Analysis of the Impact of Supply Chain Relationship Strategy on Corporate Cash Dividend Policy Based on Chinese Data

**DOI:** 10.3389/fpubh.2022.902648

**Published:** 2022-05-30

**Authors:** Xingquan Yang, Lingyan Sun

**Affiliations:** School of Economics and Management, Shihezi University, Shihezi, China

**Keywords:** supplier concentration, supplier financing, cash dividends, agency cost, financing constraints

## Abstract

With the development and application of e-commerce in the process of supply chain integration, the choice of supplier centralized strategy or decentralized mode and how to use supplier financing have become significant contents of supply chain management. This study investigates the effect of competition and cooperation on the corporate cash dividend policy under the influence of the supplier relationship strategy and its mediating mechanism based on Chinese data. The motivation of this study is to provide a basis for enterprises to grasp the dynamic evolution process of the economic consequences of supply chain relationships based on big data and adjust the relationship strategy in time to maximize the positive effects of supplier relationships. This study considers supplier concentration and supplier financing as two dimensions to measure the supplier relationship strategy and selects the balanced panel data of Chinese A-share listed companies from 2007 to 2020 as samples by applying the Logit and Tobit model. The results demonstrate that the supplier relationship is negatively correlated with the cash dividends. The intermediary effect found that the competition effect of the supplier relationship aggravates the agency conflict of enterprises and intensifies the degree of financing constraints, and thus acts on the cash dividends of enterprises. This study expands the economic consequences of relational transactions and provides an explanation of dividend policies from the perspective of a supply chain.

## Introduction

With the development of the supply chain, the influence of supplier relationship strategy formed by enterprise relationships on enterprise financial decisions has gradually attracted the attention of academic circles. There are numerous and extensive nonfinancial studies on the economic consequences of supplier relationship strategy, and the primary research contents include the following components: influence factors of the supplier relationship strategy (1); supplier relationship dimension (2); the influence of the supplier relationship on the enterprise performance (3, 4), supplier relationship integration (5, 6) supplier collaboration (7–9), information sharing (10), and the related incentive and constraint mechanisms (11–13). In recent years, the research with regard to the influence of the supplier relationship on enterprise finance aspects has also attracted the attention of scholars, which primarily involves the influence of the supplier relationship on the enterprise business credit (14–16), cash holdings (17), capital structure (18–20), transaction costs (21, 22), the choice of corporate governance (23–25), bargaining power (26), earnings management (27), financial distress, and the bankruptcy risk of enterprises (28). However, the influence of the supplier relationship strategy on cash dividend policy is rarely investigated in the extant literature. Existing studies on the impact of supply chain on shareholder wealth only discuss the perspective of customers, ignoring the impact of supplier relationships on dividends (29, 30). Actually, with the development of e-commerce, the business-to-customer model saves the cost of looking for customers and weakens the advantage of buyer's market; In contrast, there is a direct market transaction relationship between suppliers and companies, so suppliers have a greater impact on the transaction costs, investment efficiency and business decisions of enterprises than customers. Therefore, the study on how supplier relationship affects corporate financial decisions not only expands the research on the stakeholder factors of dividend policy but also provides a basis for enterprises to timely judge and adjust relevant business strategies and financial decisions based on the dynamic evolution of supplier relationship observed by big data analysis.

By summarizing the existing empirical research on cash dividend policy, scholars mainly discuss the factors affecting dividend payments from the macro and micro levels: At the micro-level, it mainly includes interest correlation (31), capital structure (32), corporate governance (33, 34), risk (35), ownership structure (36), etc. At the macro level, it mainly involves the legal environment (37), market environment (38), cultural and political factors (37), and external macro-environment (39). These factors are based on the theoretical basis of information asymmetry (40) or agency cost aspect (31, 41, 42) and the financing constraint hypothesis (43, 44). By summarizing empirical research and relevant theoretical findings, although the academic research with regard to the cash dividends has been expanding the explanation of its influencing factors from the theoretical and empirical perspectives, its underlying source can be summarized as the agency theory of enterprises and the perspective of information asymmetry. The early literature on dividend influencing factors only discussed a single aspect, until the dividend tradeoff model proved by Rozeff showed that dividend policy was the result of the joint influence of agency cost and financing constraints under information asymmetry. On the one hand, the information asymmetry between managers and shareholders causes managers to control the cash flow by reducing cash dividends at the expense of shareholders' interests, maximizing their own interests (41). Therefore, increasing cash dividends can restrain agency costs. However, the increase in the cash dividends gives rise to the higher external financing cost while restraining the agency problem, and consequently, the determination of the company's optimal dividend policy requires considering both effects. In addition, in actual corporate governance, enterprises with severe agency costs simultaneously burden financing constraints, and thus, the formulation of optimal financial decisions should be according to both agency costs and financing constraints (43). Therefore, by further expanding the theory of Rozeff (45), Chae et al. (33) conducted an empirical test by using the data of the US-listed companies and proves that the relationship between the dividend payments level and the corporate governance level depends on the relative size of agency costs and external financing costs. Based on the status quo of weak corporate governance and weak investor legal protection in China's listed companies, the problem of financing constraints and agency costs caused by the information asymmetry is serious and longstanding, which curbs corporate governance as well as financial decision making. The scholars gradually began to explore the influence of agency costs and financing constraints on the corporate cash dividend policy from the dual perspectives. Lu and Wang (34) confirmed that when companies faced agency costs and financing constraints simultaneously, companies with good corporate governance would increase the payment of the cash dividend. Subsequently, from the perspective of the cash dividend policy, Zhong and Lu (46) proved that the signal transmission of the cash dividend policy should take into account the dual restriction of financing constraints and agency costs. Yu and Liang (44) proved that China's low dividend is the result of the dual effects of agency cost and financing constraints according to the deviation degree of the actual dividend level.

The relationship between enterprises and suppliers is a dynamic process in which cooperation and competition coexist, and it is internalized into the competition and cooperation relationship. The cooperative effect of suppliers may promote information sharing through relational transactions and constitute an external supervision mechanism to increase enterprises' profitability, thus alleviating the agency cost of enterprises, and consequently, affecting cash dividends. It is also possible to increase cash dividends by easing the financing constraints of enterprises by supplying the commercial credit provided by suppliers. The competitive effect of suppliers on the cash dividend policy of enterprises is primarily manifested in the predatory practices induced by both sides of the transaction to maximize their own interests. On the one hand, the predatory practices of enterprises to suppliers are reflected by opportunism and moral hazard. Thus, the management tends to take advantage of the buyer by inducing suppliers to provide them with on-the-job consumption and personal opportunities or by facilitating participation in the board of directors, and by other covert behaviors, such as transferring corporate wealth, increasing agency costs, and reducing cash dividends. On the other hand, to maximize their own interests, suppliers with bargaining advantages rip off downstream enterprises, reduce the supply of commercial credit, increase the financing constraints of enterprises, and thus reduce cash dividends. In this scenario, this study investigates whether the supplier relationship strategy induces a significant impact on the cash dividend policy of enterprises. If the relationship exists, the central mechanism of action is attributed to the cooperative effect or competition effect.

In this study, the annual reports of companies listed from 2007 to 2020 are used to investigate the two dimensions, namely, supplier concentration and supplier commercial credit financing, and judge their impact on the corporate cash dividend policy. Subsequently, these dimensions are used to explore the mediating mechanism of supplier relationship strategy affecting cash dividends. The test results show that the supplier concentration is negatively correlated with the cash dividend payments tendency as well as the cash dividend payments level. Supplier commercial credit financing is negatively correlated with the cash dividend payments level and the cash dividend payments intention. The mediation mechanism test supports the competitive effect of the supplier relationship strategy. The supplier relationship strategy increases the agency cost of enterprises and the financing constraint of enterprises, which reduces the dividend payments tendency and the dividend payments level. It provides an explanation of the negative dividend policy based on the perspective of the supply chain.

As the rare empirical study on the impact of supplier relationship strategy on corporate cash dividends, this study explores the cash dividend policy of enterprises from the perspective of the supplier relationship strategy, which not only enriches the relevant literature concerning the influence of the supplier relationship strategy on enterprise behavior but also provides certain enlightenment for enterprises to improve the supplier relationship strategy and optimize the cash dividend policy. This study differs from Wang's (29) study that only discusses the impact of a single level of customer relationship on cash dividends. Wang (29) believes that the financial distress hypothesis and certification hypothesis are the theoretical basis for the effect of economic consequences of customer relationships on corporate cash dividends, and finally proves that the financial distress hypothesis is valid. Different from the research of Wang (29), this article from the perspective of the supplier relationship, summarizes the root causes of its economic consequences as the cooperation effect and competition effect and proves that the supplier relationship affects the agency cost and financing constraints of enterprises through competition effect, thus acting on cash dividends, which is different from the economic consequences of financial distress in customer relationships studied by Wang (29).

The contribution and research motivation of this study can be summarized as follows: First, it can provide a basis for enterprises to grasp the status of cooperation effect and competition effect of supplier relationship in time by using big data analysis, adjust financial strategy in time to improve corporate governance level and anti-risk ability, and maximize the positive effect of supplier relationship. Second, the research conclusion proves the significance of the information effect on supplier relationships, which will promote the comprehensiveness and timeliness of big data construction and provide a reference for the decision-making of regulators and investors at the supply chain level.

## Literature Review and Hypothesis Development

The supplier relationship primarily impacts the cash dividend policy of enterprises by using the “cooperation effect” and the “competition effect,” thus generating “positive” and “negative” effects, respectively.

### Supplier Relations and the Corporate Cash Dividend Policy: Cooperative Effect

The cooperative relationship is an enterprise relationship based on a long-term written contract, which can play a greater role as compared to the contract and enable both parties to share information, risks, and benefits over a long period of time (47). On the one hand, the cooperation effect of suppliers can constitute the external governance mechanism of enterprises, improve the level of corporate governance, alleviate the agency cost of enterprises, and increase the cash dividends. Specifically, the formation of supplier relationships constitutes a proprietary relationship investment between the buyer and the seller and consequently provides suppliers with access to acquire enterprise proprietary information (19, 20, 48). Accordingly, the supplier can serve as the supervision and certification entity of the enterprise and supervise the opportunistic behavior of the enterprise management, thus reducing the agency cost. In conclusion, the cooperative effect of the supplier relationship alleviates the agency problem while improving the corporate governance environment, and thus it can optimize the enterprise's production and operation activities, generate higher profitability and performance, and increase the company's ability to pay dividends, which is found to be consistent with the conclusion of the “outcome model” of dividend payments of La Porta et al. (42), that is, the company may increase the willingness and the level of the dividend payments.

On the other hand, suppliers can achieve in-depth cooperation by participating in joint research and development of enterprises, offering inventory management for downstream enterprises, or by providing trade discounts and relaxing the term of the commercial credit, which eases the financial constraints of enterprises to some extent, thus enabling enterprises to have more sufficient cash for the dividend payments (40, 49–51). In addition, supplier financing is considered an effective alternative financing instrument that enterprises can use to alleviate financing constraints when the formal system is imperfect. The alleviation of financing constraints implies reducing the cost of external funds and accordingly increasing the cash dividend payments. Based on the above analysis, Hypothesis 1 is proposed.

Hypothesis 1: The supplier relationship is positively correlated with the cash dividend payments.

### Supplier Relations and Cash Dividend Policies: Competitive Effect

In the competition, both parties will face the possibility of being misappropriated profit by the party with bargaining advantages. The competitive model of the supplier relationship acts on the corporate cash dividend policy by aggravating agency conflicts and financing constraints.

Due to different risk preferences and return functions faced by the management and shareholders, the manager has a strong advantage to retain more free cash flow based on self-serving behaviors, such as on-the-job consumption, which leads to the appropriation of the cash dividend payments level of the company (33). The subtle relationship between enterprises and suppliers appropriately provides convenient conditions for the management to retain cash. On the one hand, under the competitive effect, the management takes advantage of its buyer's market advantage and seeks to preserve the free cash flow to a greater extent under the pretext of investing in relational proprietary assets in suppliers, and thus, it provides opportunities to them to increase in-service consumption and personal empire construction and increases agency costs, and thus exacerbating the agency problem and expropriating the cash used for the dividend payments (52). In addition, under the influence of competition, suppliers are more inclined to maximize their own interests and use interests as bait to lobby the management of downstream enterprises to facilitate transactions without considering the real capabilities of suppliers. This leads to an increase in inefficient investment due to a lack of high-quality suppliers. Furthermore, the supplier shows opportunistic behavior toward the enterprise for maximizing its own interests, more specifically, exploiting the profits of downstream enterprises by offering low-quality raw materials, thus resulting in an inefficient relational asset investment, which consequently reduces the value of the company and encroaches on the cash used to pay dividends (53).

The supplier concentration degree formed by the supplier relationship reflects the bargaining advantage of suppliers, which easily leads to the competition effect (54), thus reducing the supply of the commercial credit (55). The reason is that under the circumstance of a powerful position possessed by suppliers, even if they reduce the provision of commercial credit to enterprises, they are less likely to face the risk of enterprises changing suppliers. Under the circumstance that suppliers are highly concentrated and have obvious bargaining advantages, they tend to expropriate the cash flow of enterprises by using their bargaining advantage, for instance, decreasing the supply of the commercial credit, increasing the prices of materials, reducing discounts, elevating cash sales, decreasing credit sales, and shortening the payback periods of goods, and thus increasing the degree of financial constraints of enterprises (55). Consequently, it encroaches on the cash quota of cash dividends issued by enterprises and reduces the tendency and the payment intensity of cash dividends (56). Based on the above analysis, Hypothesis 2 is proposed.

Hypothesis 2: The supplier relationship is negatively correlated with the cash dividend payments.

## Sample Selection and Data

### Sample Selection

In this study, the data of Chinese A-share listed companies from the period 2007 to 2020 were selected as initial research samples; all details were taken from the “CSMAR” database and WIND database. In addition, for the supplement of missing values, this study searched its annual report through the website of Oriental Fortune and big data and calculated its missing indicators according to the index calculation formula. In this study, the sample companies with excessive outliers and unavailable values are excluded, whereas for the companies with less unavailable values, the annual reports were searched online, and the unavailable values were calculated according to the index calculation formula. To improve the accuracy of the empirical results, the data were analyzed based on the following steps: in view of the current situation of financial anomalies or continuous losses of ST-class and PT-class listed companies, liquidity constraints were very serious. Hence, the samples of such companies were first excluded. Given that the characteristics of capital expenditure in the financial service industries are vastly different from those of other firms, banks, insurance companies, securities, and other types of companies were excluded, simultaneously, companies with incomplete data on relevant indicators, such as corporate governance, were eliminated. To reduce the estimation error caused by statistical errors or abnormal samples and to consider the influence of extreme values, all data were Winsorize processed at 1% and 99% quantiles.

### Model Specification

According to the characteristics of the explained variables and sample data, this study uses the Logit model (1) of panel data to study the influence of the supplier concentration/supplier financing on the cash dividend payment tendency of the listed companies. In cases when some enterprises do not pay cash dividends, the ratio of the cash dividend is 0. This makes the cash dividend payment rate a trailing variable with a lower limit of 0. If the regression method of the ordinary OLS is used, it becomes easy to produce deviation. As a consequence, this study refers to the study of Fama and French (57), Brockman and Unlu (31, 32) to develop the Logit model (1) and the Tobit model (2) of the dividend payments. In models (1) and (2), the subscript *i* represents the company, *t* represents the year, the explanatory variable top 5 represents the supplier concentration, credit represents the supplier credit financing, and controls represent a group of control variables. The Logit model and the Tobit model are explained as follows.

Logit model: It takes into consideration the supplier concentration, supplier commercial credit financing, and cash dividend payment tendency:


(1) $$\begin{array}{l} L\text{ogit}(d\_payeri,t)={\text{α}}_{\text{0}}+{\text{α}}_{\text{1}}Top5/credit_{i,t}+\Sigma {\text{α}}_{2}control\\ \;+\Sigma Year+\Sigma Ind+\varepsilon_{i,t} \end{array}$$

Tobit model: It considers the supplier concentration, supplier commercial credit financing, and cash dividend payment level:


(2) $$\begin{array}{l} \text{Tobit}(div\text{i-}ratio,t)=\beta_{0}+\beta_{\text{1}}Top5/credit_{i,t}+\Sigma \beta_{2}control\\ \;+\Sigma Year+\Sigma Ind+\varepsilon_{i,t} \end{array}$$

### Definition of Variables

#### Explained Variable

Considering the discrepancy between the cash dividend payment tendency and the cash dividend payment degree, this study refers to the research of Denis and Osobov (58) and uses the cash dividend payment tendency (d_payer) and the payment level (divi-ratio) to measure the cash dividend policy of the listed companies. Specifically, the cash dividend payment tendency (d_payer) is considered as the dummy variable. If the enterprise pays the cash dividends in the current year, d_payer is considered as 1; otherwise, it is considered as 0. The cash dividend payout level (divi-ratio) is calculated as the ratio of cash dividends to net income.

#### Explaining Variable

To fully obtain the cooperation and competition status of the supplier relationship strategy, the measurement index of the supplier relationship strategy requires reflecting not only the closeness of the relationship between enterprises and suppliers but also their bargaining advantages. This study refers to the existing literature and applies supplier concentration as one of the indicators to measure the supplier relationship strategy (16, 17, 59, 60). For another dimension of supplier relations, considering the work of Wang and Wang (61), supplier financing that is the net occupation of enterprises, to supplier financing, is selected.

(a) Supplier concentration (Top5): For the measurement of the supplier relationship strategy, most of the earlier studies have used the supplier concentration degree as a proxy. A high proportion of the Top5 implies a close supplier relationship and a high bargaining advantage for suppliers. In this study, the proportion of the purchasing amount of the top five suppliers in the total annual purchasing amount of the listed companies is used as the measure of the supplier concentration.

(b) Supplier commercial credit financing (credit): The existing literature considers that supplier commercial credit financing is the exclusive asset investment existing in the transaction of the supplier relationship strategy, which suggests the higher the proportion of enterprises' commercial credit financing with suppliers, the closer the relationship with suppliers and the stronger the bargaining advantage gained by suppliers. This study refers to the measurement method of the utilization level of supplier financing proposed by Wang and Wang (61), which is measured as the proportion of the enterprise's net occupation of suppliers to the cost of sales. The larger the proportion of the enterprise's net occupation of suppliers, the more the supplier financing, and hence the indicator is calculated as (accounts payable + notes payable – accounts prepaid)/cost of sales.

#### Other Control Variables

In addition, the model also controls other factors that affect dividend payments. Refer to the studies of Chae et al. (33) and Brockman and Unlu (31), Profit, growth, ROE, age, and state are selected as control variables in this study, and the calculation method is as follows: Profit, that is, the ratio of net profit to total assets. The growth of the company is expressed by the growth rate of the main business income. ROE refers to return on equity, and the company's listing age is the same year —- the company's listing year +1, and the property rights of the company (State). In addition, the control variables also included the industry dummy variable Ind and the annual dummy variable Year.

## Empirical Evidence

### Descriptive Statistics

Table 1 presents the descriptive statistical characteristics of the main variables. From the descriptive statistics, it can be observed that the mean value of d_payer for the dividend payments is 0.711, which indicates that more than 70% of the sample listed companies have paid cash dividends. The mean value of supplier concentration is 24.3%, which indicates that China's listed companies show a relatively low degree of supplier concentration. In addition, the average ratio of enterprise supplier financing to the cost of sales (credit) is measured as 26.3%, with a maximum value of 146.1% and a minimum value of −47.9%. It can be concluded that Chinese A-share listed companies have great differences in the capital occupation of suppliers.

**Table 1:** Descriptive statistics.

**VarName**	**Obs**	**Mean**	**SD**	**Min**	**Median**	**Max**
dpayer	34,237	0.711	0.453	0.000	1.000	1.000
divi-ratio	35,005	0.275	1.033	−64.428	0.204	107.407
divi-ratio2	34,459	0.116	0.357	0.000	0.014	2.623
Age	34,459	10.367	7.058	1.000	9.000	27.000
Growth	35,005	0.152	0.338	−0.591	0.108	1.844
Profit	35,005	3.999	6.322	−26.552	3.920	21.131
ROE	35,005	0.077	0.128	−0.573	0.079	0.419
State	35,005	0.397	0.489	0.000	0.000	1.000
Top 5	35,005	0.243	0.235	0.000	0.209	0.943
Credit	35,005	0.263	0.282	−0.479	0.209	1.461

### Basic Regression Results of the Supplier Relationship Strategy and the Enterprise Cash Dividends

Table 2 presents the regression results of the main effects of models (1) and (2), that is, the regression result of the relationship between supplier concentration/supplier financing, dividend payout tendency, and dividend payout rate. Columns (1) and (2) of the table represent the relationship between the supplier concentration(top5)/supplier financing credit(credit) and the cash dividend payment tendency, respectively. As can be observed from column (1), the coefficient of the supplier concentration Top5 and the dividend payment tendency is −0.749, which is significant at the level of 1%, indicating that the supplier concentration degree is negatively correlated with the dividend payment tendency. From the results of column (2), it can be concluded that the correlation between the supplier financing credit and the dividend payments preference d_payer is negative. Columns (4) and (5) of the table show the Tobit regression results of the model (2), namely, the relation between supplier concentration top5/supplier financing credit and the cash dividend payment level. As can be observed from column (4), the coefficient between the supplier concentration and the dividend payment level is −0.065, which is significant at the level of 1%. From column (5) of the table, it can be concluded that the coefficient of supplier credit financing and dividend payment intensity is significantly negative at the level of 1%, with a coefficient of −0.074. Therefore, the supplier concentration degree/supplier financing is negatively correlated with the cash dividend payment level, which proves Hypothesis 2.

**Table 2:** Supplier relationship strategy top5/credit and dividend payout propensity (logit), dividend payout level (Tobit) regression results.

	**(1)**	**(2)**	**(3)**	**(4)**	**(5)**	**(6)**
	**d_payer**	**d_payer**	**d_payer**	**divi-ratio**	**divi-ratio**	**divi-ratio**
Age	−0.092***	−0.091***	−0.093***	−0.011***	−0.011***	−0.012***
	(−36.05)	(−35.61)	(−36.22)	(−30.34)	(−30.13)	(−30.54)
Growth	−0.113**	−0.131***	−0.118***	−0.097***	−0.099***	−0.098***
	(−2.51)	(−2.92)	(−2.62)	(−13.34)	(−13.62)	(−13.49)
Profit	0.204***	0.197***	0.200***	0.019***	0.018***	0.018***
	(29.63)	(28.84)	(29.07)	(24.45)	(23.08)	(23.21)
ROE	4.487***	4.721***	4.577***	0.324***	0.355***	0.344***
	(14.69)	(15.49)	(15.01)	(8.49)	(9.25)	(8.97)
State	0.396***	0.416***	0.399***	0.021***	0.023***	0.022***
	(11.49)	(12.10)	(11.59)	(3.87)	(4.30)	(3.98)
Top5	−0.749***		−0.776***	−0.065***		−0.070***
	(−10.51)		(−10.85)	(−5.84)		(−6.32)
Credit		−0.353***	−0.385***		−0.074***	−0.077***
		(−6.24)	(−6.79)		(−8.22)	(−8.57)
_cons	−0.835***	−0.817***	−0.843***	0.081**	0.082**	0.081**
	(−4.25)	(−4.16)	(−4.30)	(2.37)	(2.39)	(2.37)
ind	Yes	Yes	Yes	Yes	Yes	Yes
yr	Yes	Yes	Yes	Yes	Yes	Yes
*N*	34,237	34,237	34,237	34,459	34,459	34,459

^*^is significant at 0.1 level, ^**^is significant at 0.05 level, ^***^is significant at 0.01 level (the same below).

### Robustness Test

The above empirical results may be affected by quantitative problems, such as inverse causality problems, missing variables, and sample selection bias. Therefore, this study performed the following robustness test to ensure the reliability of the above conclusions.

#### Reverse Causality Problem

The selection of the dividend policy may affect the enterprise's supplier concentration and supplier financing. In other words, there is a potential reverse causality between the supplier relationship strategy and the cash dividend payment level. To test this problem, this study considers the dividend payment tendency of the next phase *dpayer*_t+1_ and the cash dividend payment level of the next phase *divi*−*ratio*_t+1_ as the explained variables. The regression results are presented in Table 3. The results in column (1) prove that there is a significant negative correlation between the supplier concentration (top5) and the cash dividend payment level of the next phase at the level of 1%. In addition, based on the results presented in column (3), the supplier concentration is negatively correlated with the dividend payment tendency of the next phase, which is found to be consistent with the above conclusion. Similarly, based on the results presented in column (2), it is proved that supplier commercial credit financing is significantly negatively correlated with the cash dividend payment level of the next phase, which is found to be consistent with the above conclusion. The results of column (4) also prove the robustness of the results.

**Table 3:** Inverse causality problem test and the substitution key variable method.

	**Inverse causality problem test**				**Substitution key variable method**	
	**(1)**	**(2)**	**(3)**	**(4)**	**(5)**	**(6)**
	**divi-ratiot+1**	**divi-ratiot+1**	**dpayert+1**	**dpayert+1**	**divi-ratio**	**divi-ratio2**
Age	−0.010***	−0.010***	−0.076***	−0.075***	−0.012***	−0.002***
	(−24.85)	(−24.64)	(−29.65)	(−29.31)	(−30.54)	(−6.54)
Growth	−0.087***	−0.089***	−0.084*	−0.099**	−0.098***	−0.052***
	(−11.05)	(−11.30)	(−1.90)	(−2.24)	(−13.49)	(−7.86)
profit	0.018***	0.017***	0.131***	0.126***	0.018***	0.008***
	(21.65)	(20.43)	(22.30)	(21.50)	(23.21)	(11.16)
ROE	0.318***	0.346***	3.915***	4.075***	0.344***	1.072***
	(7.78)	(8.44)	(14.37)	(14.93)	(8.97)	(28.00)
State	0.020***	0.023***	0.354***	0.370***	0.022***	0.073***
	(3.50)	(3.88)	(10.41)	(10.89)	(3.98)	(14.79)
Top5	−0.060***		−0.577***		−0.070***	−0.223***
	(−4.96)		(−8.19)		(−6.32)	(−21.96)
Credit		−0.075***		−0.353***	−0.077***	−0.063***
		(−7.62)		(−6.19)	(−8.57)	(−7.70)
_cons	−0.021	−0.019	−0.831***	−0.815***	0.081**	−0.319***
	(−0.58)	(−0.53)	(−4.32)	(−4.24)	(2.37)	(−10.05)
ind	Yes	Yes	Yes	Yes	Yes	Yes
yr	Yes	Yes	Yes	Yes	Yes	Yes
*N*	30,629	30,629	30,409	30,409	34,459	34,459

^*^is significant at 0.1 level, ^**^is significant at 0.05 level, ^***^is significant at 0.01 level (the same below).

#### Replace Key Variables

To ensure the robustness of the results, this study refers to Kao and Chen (38) and Xu and Xu (62) to replace the measurement index of dividend payment level. As a consequence, this study uses the cash dividend yield divi-ratio2, which is calculated as the ratio of the cumulative cash dividend paid to the closing price of the year substituted for the dividend payment level. The results are presented in columns (5) and (6) of Table 3. Column (5) shows the regression results of the relationship between the supplier concentration top5/supplier commercial credit financing and the original variable divi-ratio. Column (6) shows a significant relationship between top5, credit, and the substitution variable divi-ratio2, indicating that the result is still negatively correlated at the level of 1%, which is found to be consistent with the previous conclusion, thus proving the robustness of the results.

#### Propensity Score Matching (PSM)

Since the model may exist as the endogeneity of missing variables, it may be related to both the dividend payments and the supplier relationship strategy. Specifically, if the control variables in the model fail to well capture the discrepancies between the centralized and decentralized suppliers, as well as the diversities in the characteristics of the supplier financing scale, the measurement index of the supplier relationship strategy will induce a nonlinear effect. Due to the special types of the Logit and Tobit models, this study uses the propensity score matching method to control the endogeneity of missing variables. This study refers to the research methods proposed by Jiao and Zhang (30) and Meng and Bai (63). The treatment and control groups were divided considering the fact whether the quantile of the Top5 of the enterprise was >60% of the sample (60% quantile of the Top5 and credit variables were used to keep the result unchanged). The propensity score was calculated by using Logit regression. The asset size LNsize, financial leverage Lev, sales revenue Growth rate, property right property State, company Age, market value to the book value ratio Mkt, and industry HHI were used as matching variables. Subsequently, the propensity score matching analysis was performed based on the data of the enterprise in the current year. The model used the most common method of “nearest-neighbor matching” to match the treatment and the control groups. The results are presented in Table 4. In column (13) of the table, the *P* values are all >0.05. After matching all variables, no significant difference was observed between the treatment and the control groups, indicating that there is no statistical difference between them. Moreover, the mean difference between the groups is not found to be significant, which basically satisfies the balance test of PSM. In this study, after the PSM test, the results are presented in Table 5. Comparing this result with the main effect result presented in Table 2, it is found that in column (1) of Table 5, for the Tobit model of the dividend payment level, the regression coefficient of Top5 is −0.072. The regression coefficients of the top5 were found to be significantly negatively correlated, which proves the robustness of the results. Similarly, the regression coefficients of credit in column (1) of Table 5 demonstrate the robustness of the results.

**Table 4:** Average processing effect of PSM (ATT).

**(1) Variable**	**(2) Sample**	**(3) Treated**	**(4) Controls**	**(5) Difference**	**(6) S.E**	**(7) T-stat**
divi-ratio	Unmatched	0.273659	0.27030	0.00335	0 0.0095	0.35
	ATT	0.273699	0.29912	−0. 02542	0.0158	−1.60**
Variable	Sample	Treated	Controls	Difference	S.E	T-stat
d_payer	Unmatched	0.7157	0.7048	0.0109	0 0.0050	2.18**
	ATT	0.7159	0.7244	−0.0085	0.0068	−1.24*
**(8)** **Variable**	**(9)** **Treated**	**(10)** **control**	**(11)** **%bias**	**(12)** ***t***	**(13)** ***p*****>|t|**	**(14)** **V (T)/V (C)**
Age	10.031	10.128	−1.4	−1.11	0.269	1.23*
Growth	0.1510	0.1473	1.1	0.89	0.371	1.29*
Lev	0.3984	0.4067	−0.2	−1.9	0.057	0.72*
Mkt	2.618	2.816	−1.1	−0.8	0.423	0.02*
State	0.3233	0.3152	1.7	1.44	0.150	
HHI	0.1066	0.10638	0.2	0.21	0.833	1.09*
LNSize	21.838	21.858	1.9	1.66	0.097	0.96*

*is significant at 0.1 level, **is significant at 0.05 level, ***is significant at 0.01 level (the same below).

**Table 5:** Propensity score matching (PSM processing).

	**(1)**	**(2)**
	**divi-ratio**	**d_payer**
Age	−0.011***	−0.092***
	(−30.14)	(−35.89)
Growth	−0.099***	−0.114**
	(−13.55)	(−2.53)
profit	0.018***	0.202***
	(22.59)	(29.05)
ROE	0.338***	4.534***
	(8.85)	(14.76)
State	0.019***	0.396***
	(3.50)	(11.44)
Top5	−0.072***	−0.782***
	(−6.49)	(−10.90)
Credit	−0.077***	−0.382***
	(−8.55)	(−6.69)
_cons	0.087**	−0.862***
	(2.53)	(−4.39)
ind	Yes	Yes
yr	Yes	Yes
*N*	34,070	34,070

^*^is significant at 0.1 level, ^**^is significant at 0.05 level, ^***^is significant at 0.01 level (the same below).

## Intermediary Mechanism Test of the Supplier Relationship Strategy and the Cash Dividend Payment Level

The intermediary mechanism model can analyze the process and mechanism of the influence of independent variables on dependent variables. This study argues that supplier concentration and supplier financing act on corporate cash dividends through agency costs and financing constraints. The most popular causal steps approach for testing is the Baron and Kenny (64) causal steps approach and the Sobel (65) approach. Therefore, by combining the advantages of the above methods, this study uses the mediating effect analysis method summarized by Baron and Kenny (64) and Wen and Ye (66) to examine the influence mechanism of supplier relationship strategy on cash dividends.

### Mediation Variables and Model Specification

Mediation variables: The agent cost AC and financing constraint FC are considered as mediation variables. This study refers to the existing literature. For the measurement of the first type of agency cost, this study refers to the approaches proposed by Singh and Davidson III (36), Li (67) and Jiang et al. (68). In this study, the management expense ratio AC is used to measure the first type of agency cost, and the calculation method is measured as the ratio of the management expense to the main business income. This study exploits the SA index developed by Hadlock and Pierce (69) to measure the degree of the financing constraint (FC) of the listed companies. SA = −0.737 × company size + 0.043 ^*^ square of the total assets −0.04 × company listing time. The larger the SA index, the smaller the degree of the financing constraint. To test the effect mechanism of the supplier relationship strategy on the corporate cash dividends, this study uses the mediating effect analysis method proposed by Baron and Wen, and this specific model is expressed as follows:


(3a) $$\begin{array}{l} AC={\text{χ}}_{0}+{\text{χ}}_{\text{1}}Top5/credit_{i,t}++\Sigma {\text{χ}}_{2}control+\Sigma Year\\ \;+\Sigma Ind+\varepsilon_{i,t} \end{array}$$

(3b) $$\begin{array}{l} FC=\gamma_{0}+\gamma_{1}Top5/credit_{i,t}++\Sigma \gamma_{2}control+\Sigma Year\\ \;+\Sigma Ind+\varepsilon_{i,t} \end{array}$$

(4a) $$\begin{array}{l} T\text{obit}(\text{d}ivi-ratio_{i,t})/\log it(d\_\text{payer})=\varphi_{0}+\varphi_{1}Top5/credit_{i,t}\\ \;+\;\varphi_{2}AC+\sum \varphi_{3}control\text{s}+\Sigma Year+\Sigma Ind\\ \;+\varepsilon_{i,t} \end{array}$$

(4b) $$\begin{array}{l} T\text{obit}(\text{d}ivi-ratio_{i,t})/\log it(d\_payer)=\delta_{0}+\delta_{1}Top5/credit_{i,t}\\ \;+\delta_{2}FC+\sum \delta_{3}control\text{s}+\Sigma Year+\Sigma Ind\\ \;+\varepsilon_{i,t} \end{array}$$

### Empirical Results of the Mechanism Test

Table 6 presents the results of the mechanism test for the relationship between the supplier concentration and the cash dividend payment preference d_payer as well as the cash dividend payment level divi-ratio, respectively. The results show that the supplier concentration affects the willingness and the level of the cash dividend payments by increasing the agency cost and the financing constraint. The specific testing steps followed in the intermediary effect mechanism are the following.

**Table 6:** The mechanism test of supplier concentration acts on cash dividend payment tendency and dividend payment level.

	**(1)**	**(2)**	**(3)**	**(4)**	**(5)**	**(6)**
	**AC**	**divi-ratio**	**d_payer**	**fc**	**divi-ratio**	**d_payer**
Age	0.001***	−0.013***	−0.095***	−0.004**	−0.013***	−0.014***
	(3.04)	(−17.70)	(−19.51)	(−2.29)	(−17.70)	(−22.21)
Growth	−0.025***	−0.108***	−0.398***	0.101***	−0.106***	−0.055***
	(−5.47)	(−11.03)	(−6.99)	(5.07)	(−10.85)	(−6.74)
Profit	0.005***	0.022***	0.340***	−0.040***	0.025***	0.032***
	(5.82)	(12.25)	(19.92)	(−9.49)	(13.62)	(22.01)
ROE	−0.402***	0.070	0.691	2.948***	−0.065	0.251***
	(−10.57)	(0.79)	(0.97)	(13.97)	(−0.72)	(3.64)
State	0.018***	−0.033***	−0.324***	−0.377***	−0.019**	−0.013
	(3.79)	(−3.50)	(−5.32)	(−19.38)	(−2.00)	(−1.58)
Top5	0.023***	−0.042***	−0.573***	−0.600***	−0.016	−0.038***
	(3.54)	(−3.15)	(−6.82)	(−21.39)	(−1.19)	(−3.19)
AC		−0.446***	−3.507***			
		(−7.69)	(−9.06)			
FC					0.055***	0.085***
					(11.38)	(20.27)
_cons	0.085***	0.323***	2.296***	−0.095	0.243***	0.802***
	(3.15)	(6.35)	(6.91)	(−0.93)	(4.79)	(17.97)
ind	Yes	Yes	Yes	Yes	Yes	Yes
yr	Yes	Yes	Yes	Yes	Yes	Yes

^*^is significant at 0.1 level, ^**^is significant at 0.05 level, ^***^is significant at 0.01 level (the same below).

Step 1: Implement the regression test for the main effect, namely, models (1) and (2). The regression results are presented in Table 2, and the coefficient is found to be significantly negative, which indicates that the supplier concentration is significantly negatively correlated with the cash dividend payment tendency and the cash dividend payment level of enterprises.

Step 2: Regression is performed on models (3a) and (3b), that is, the intermediary variables (agency cost AC and financing constraint FC) were respectively regression with the supplier concentration (top5) of the explanatory variable. The results are presented in columns (1) and (4) of Table 6. As can be observed from the table, the higher the concentration of suppliers, the higher the agency cost of the enterprise would be (the coefficient between top5 and AC is measured as 0.023, which is significantly positive). At the same time, the high concentration of suppliers will increase the degree of the financing constraint of enterprises (the coefficient of top5 and FC is measured as −0.600, which is significantly negative, and the dependent variable FC represents the degree of the financing constraint; the higher the FC, the lower the degree of the financing constraint). Therefore, top5 is found to be positively correlated with the degree of financing constraint.

Step 3: For models (4a) and (4b), the explained variable (divi-ratio and d_payer), the explanatory variable (supplier concentration (top5)), and the intermediary variable (agent cost AC and financing constraint FC) were used in the same model for regression to test the significance and the positive or the negative value of the coefficient. The agent cost AC is considered as the first intermediary variable, whose coefficients with the cash dividend payment level and the payment tendency are calculated as −0.446 and −3.507 in columns (2) and (3), respectively. The coefficient is significantly negative, which indicates that the agency cost is negatively correlated with the cash dividends and the cash dividend payment tendency. This finding is consistent with the agency cost theory of dividends. Similarly, for the financing constraint of the second intermediary variable, FC, whose coefficients with the cash dividend payment level and the payment tendency are 0.055 and 0.085 in columns (5) and (6), the significance is positive. Since FC represents the degree of the financing constraint, the lower the FC index, the higher the degree of the enterprise financing constraint would be. Therefore, it can be assumed that the financing constraint is negatively correlated with the cash dividend payment level and the payment tendency, which is found to be consistent with the financing constraint theory of dividends, that is, enterprises with a higher financing constraint level show a lower cash dividend payment level.

Step 4: It presents the validation of the mediating effect according to the significance of the coefficient and its symbol. When the dividend payment level (divi-ratio) and the dividend payment preference (d_payer) are considered as the explained variables, based on the judgmental criteria of the intermediary effect, the agent cost AC is selected as an example. According to the results that the coefficients β_1_, α_1_, χ_1_, φ_1_, and φ_2_ are all significant, top5 is found to be significantly negatively correlated with the dividend payment level and the dividend payment tendency, as presented in columns (2) and (3) of Table 6, which shows the partial intermediary effect of the agency cost on the relationship between the supplier concentration and the cash dividends. The intermediary effect of the agency cost and the financing constraint on the relationship between the supplier concentration and the cash dividends is explained as follows: combining the results of models (1), (2), (3a), and (4a), the following results can be obtained: the coefficient of the explanatory variable top5 in model (3a) is measured as 0.023, and its product with the coefficient of the agent cost AC in model (4a) is found to be negative, i.e., −0.446, which is the same as the negative sign of the coefficient of the explanatory variable top5 in model (4a), i.e., −0.042. This result indicates that part of the intermediary effect of the agent cost exists, and the total proportion of the intermediary effect is determined as 0.023 ^*^ (−0.446)/(−0.042) = −0.24. Similarly, the partial mediating effect of the agency cost on the relationship between top5 and dividend payment tendency can also be proved. The proportion of the agency cost on the mediating effect of the dividend payment tendency is 0.023 ^*^ (−3.507)/ −0.573 = 0.14. Similarly, the proportion of the mediating effect of the financing constraint to the total effect of the cash dividend payment level is determined as −0.600 ^*^ 0.055/(−0.016) = 2.0625, whereas the total effect of the cash dividend payout tendency is determined as −0.600^*^0.085/−0.038 = 1.34. To sum up, the increase in supplier concentration leads to an increase in agency costs and financing constraints of enterprises, thus reducing cash dividends.

Similarly, Table 7 presents the regression results of the mechanism of the supplier commercial credit and cash dividend payment dynamics. In Table 7, the supplier commercial credit (credit) is considered as the explanatory variable, and the dividend payment level (divi-ratio) is considered as the explained variable. The steps of the intermediary effect test of the supplier commercial credit financing and cash dividend payment level are also the same as discussed in the previous section, and the corresponding results are presented in Table 7. Based on the principle of the intermediary effect test, the mediating effect of agency cost on supplier financing, incentive payment intensity, and dividend payment tendency is 0.404 and 0.77, respectively. In particular, because the sign symbols are different, financing constraint has suppressing effects on supplier financing and dividend payment level and dividend payment tendency, which are 0.065 and 0.138, respectively.

**Table 7:** Supplier financing and cash dividend payment level mechanism test.

	**(1)**	**(2)**	**(3)**	**(4)**	**(5)**	**(6)**
	**AC**	**divi-ratio**	**d_payer**	**fc**	**divi-ratio**	**d_payer**
Age	0.001***	−0.013***	−0.095***	−0.003*	−0.013***	−0.094***
	(3.24)	(−17.76)	(−19.48)	(−1.95)	(−17.84)	(−18.82)
Growth	−0.024***	−0.109***	−0.422***	0.081***	−0.107***	−0.457***
	(−5.28)	(−11.21)	(−7.43)	(3.94)	(−10.98)	(−7.87)
Profit	0.006***	0.021***	0.329***	−0.041***	0.024***	0.374***
	(6.85)	(11.43)	(19.09)	(−9.11)	(12.82)	(21.18)
ROE	−0.428***	0.126	1.149	3.144***	−0.029	−0.677
	(−11.27)	(1.41)	(1.59)	(14.14)	(−0.32)	(−0.95)
State	0.020***	−0.035***	−0.343***	−0.395***	−0.020**	−0.157**
	(4.22)	(−3.76)	(−5.63)	(−19.41)	(−2.13)	(−2.50)
Credit	0.068***	−0.072***	−0.303***	0.108***	−0.092***	−0.569***
	(8.25)	(−4.21)	(−2.82)	(3.10)	(−5.43)	(−5.22)
AC		−0.428***	−3.453***			
		(−7.36)	(−8.91)			
FC					0.056***	0.731***
					(11.98)	(20.86)
_cons	0.072***	0.337***	2.171***	−0.401***	0.281***	1.681***
	(2.67)	(6.59)	(6.56)	(−3.99)	(5.52)	(4.87)
ind	Yes	Yes	Yes	Yes	Yes	Yes
yr	Yes	Yes	Yes	Yes	Yes	Yes

^*^is significant at 0.1 level, ^**^is significant at 0.05 level, ^***^is significant at 0.01 level (the same below).

**Figure 1:**
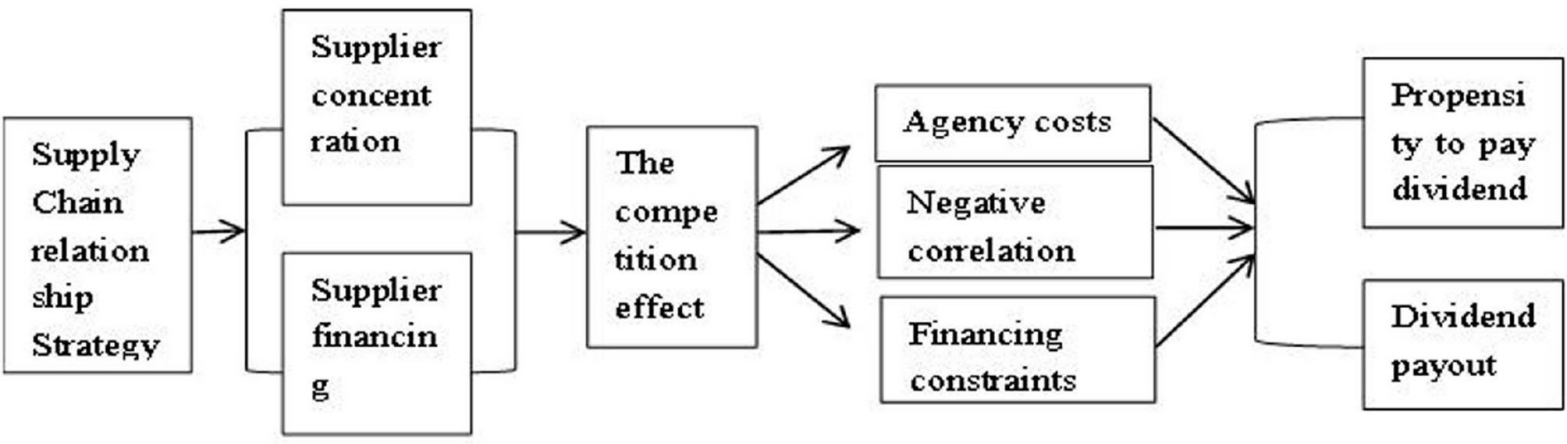
Relationship and mechanism of supplier relationship strategy and cash dividend.

To sum up, the supplier concentration degree/supplier financing primarily affects the cash dividend payment tendency and the cash dividend payment level by increasing the agency cost and the financing constraint degree. To more clearly present the relationship between supplier relationship strategy and cash dividends as well as the intermediary mechanism, the relationship between the above variables is sorted out in Figure 1, and the mediating effect of each component is shown in Table 8.

**Table 8:** Results of intermediary effect between supplier relationship strategy and cash dividend: Intermediary effect of agency cost and financing constraint.

**Intermediate effect**	**Cash dividend payment level**				**Cash dividend payout tendency**			
	**Agency cost**	**Type of intermediate effect**	**Financing constraints**	**Type of intermediate effect**	**Agency cost**	**Type of intermediate effect**	**Financing constraints**	**Type of intermediate effect**
Supplier concentration	0.24	Partial intermediate effect	2.0625	Complete mediation effect	0.14	Partial intermediate effect	1.34	Partial intermediate effect
Supplier financing	0.404	Partial intermediate effect	0.065	Suppressing effect	0.77	Partial intermediate effect	0.138	Suppressing effect

## Conclusion

The relationship transaction is increasingly affecting corporate governance and financial management. Supplier relationship strategy, i.e., choosing a centralized supplier strategy or decentralized supplier strategy and how to leverage the right supplier financing scale, has theoretical and practical significance on whether and how to influence corporate cash dividends. More importantly, the characteristic of the supplier relationship strategy is that the appearance of cooperation and competition alternately results in the various elusive economic consequences or they coexist. Therefore, enterprises need to timely judge the different economic consequences under the state of dynamic supplier relationship based on big data and timely adjust the operation and financial decisions. This study explores how the cooperative effect and competition effect produced by supplier relationship affect corporate cash dividends. It is demonstrated that supplier concentration and supplier financing, are negatively correlated with the cash dividends. Consequently, the competitive effect of the supplier relationship is proved. In the test of the intermediary mechanism of the influence of the supplier relationship strategy on the cash dividends, it is observed that the competitive effect of the supplier relationship strategy aggravates the agency conflict and the financing constraint of enterprises, and thus shows a negative effect on the willingness and level of the cash dividend payments. Based on Chinese data analysis, this study provides a decision-making basis for enterprises to maximize the positive effects of supply chain relationship strategy, and provides information basis for regulators and external investors to make decisions at the supply chain level.

## Data Availability Statement

The original contributions presented in the study are included in the article/supplementary material, further inquiries can be directed to the corresponding author.

## Author Contributions

LS contributed to methodology and writing of the article, software, data, and writing-original draft. XY contributed to reviewing and editing the article. Both authors contributed to the article and approved the submitted version.

## Funding

XY acknowledges the National Natural Science Foundation of China (No. 72062027).

## Conflict of Interest

The authors declare that the research was conducted in the absence of any commercial or financial relationships that could be construed as a potential conflict of interest.

## Publisher's Note

All claims expressed in this article are solely those of the authors and do not necessarily represent those of their affiliated organizations, or those of the publisher, the editors and the reviewers. Any product that may be evaluated in this article, or claim that may be made by its manufacturer, is not guaranteed or endorsed by the publisher.
